# Prevalence of and risk factors associated with HIV, Herpes Simplex Virus-type 2, *Chlamydia trachomatis* and *Neisseria gonorrhoeae* infections among 18–24 year old students attending Higher Learning Institutions in Mbeya-Tanzania

**DOI:** 10.1371/journal.pone.0266596

**Published:** 2022-05-26

**Authors:** Ruby Doryn Mcharo, Abisai Kisinda, Lilian Njovu, Miri Mcharo, Florida Mbwilo, Getrude Mihale, Beatrice Komba, Ernest Andrew, Philippe Mayaud, Arne Kroidl, Olena Ivanova, Sia Emmanueli Msuya

**Affiliations:** 1 National Institute for Medical Research-Mbeya Medical Research Centre (NIMR-MMRC), Mbeya, Tanzania; 2 Department of Epidemiology & Biostatistics, Institute of Public Health, Kilimanjaro Christian Medical University College (KCMUCo), Moshi, Tanzania; 3 London School of Hygiene and Tropical Medicine (LSHTM), London, United Kingdom; 4 Division of Infectious Diseases and Tropical Medicine, Medical Centre of the University of Munich (LMU), Munich, Germany; 5 German Center for Infection Research (DZIF), Partner Site Munich, Munich, Germany; 6 Community Health Department, KCMC Hospital, Moshi, Tanzania; 7 Department of Community Health, Institute of Public Health, Kilimanjaro Christian Medical University College (KCMUCo), Moshi, Tanzania; National University of Singapore, SINGAPORE

## Abstract

**Background:**

Sexually transmitted infections (STIs) are common among young people in low- and middle-income countries and are associated with negative reproductive and pregnancy outcomes. Most of the studies have assessed HIV among adolescents and young adults, with limited information on occurrence of other STIs in this population. This study aimed to describe the prevalence of and risk factors associated with Herpes Simplex Virus-type 2 (HSV-2), *Chlamydia trachomatis* (CT), *Neisseria gonorrhoeae* (NG), Syphilis and HIV infection among young adults attending Higher Learning Institutions (HLIs) in Mbeya, Tanzania.

**Methods:**

We conducted a cross-sectional study among students aged 18-24years attending HLIs in Mbeya-Tanzania, randomly selected using a computerized random number. Participants were tested for HSV-2, CT, NG, Syphilis and HIV infection. We used a self-administered questionnaire to collect information on sexual activity and risk factors to the tested STIs.

**Results:**

We enrolled 504 students from 5 HLIs, with mean age of 21.5 years (SD 1.7). 17% of the students had at least one STI; prevalence was higher among females than males (21.1% versus 14.1%). CT (11%) and HSV-2 (6.1%) were the most common STIs, while NG (1.1%) and HIV (0.7%) infection had the least occurrence. None of the participants was diagnosed with Syphilis. In univariate analysis, predictors for STIs were Sex, inconsistent condom use in the past 4weeks, report of oral sex, sexual orientation (bisexual/homosexual) and having a sexual partner with an age-difference of at least 5years (either older or younger); while in the multivariate analysis, Sex, inconsistent condom use in the past 4weeks and sexual orientation (bisexual/homosexual) remained significant.

**Conclusion:**

STIs such as Chlamydia and HSV-2 which are commonly asymptomatic are of concern among young adults attending HLIs. The latter is an important group that needs attention and recognition that is pivotal in transmission of STIs considering their risk. Information, Education and Communication (IEC) campaigns targeting young adults, especially those at HLIs, need to focus on exposure-risk minimization. Funding institutions that have invested heavily on HIV prevention campaigns should consider giving similar recognition to other STIs for a streamlined outcome.

## Introduction

Sexually transmitted infections (STIs) are a significant part of the global burden of diseases with impact on sexual and reproductive health (SRH) worldwide [1]. A number of bacteria and viruses have been shown to cause STIs with severe SRH complications. The World Health Organization (WHO) estimated that in 2020 there were 374 million new infections with one of the four curable STIs–Chlamydia, Gonorrhoeae, Syphilis and Trichomoniasis [1]. Burden of viral STIs is high with Herpes Simplex Virus (“*herpes*”, HSV), Human Papilloma Virus (HPV) and HIV affecting more than 490 million people, 300 million women and about 38 million people worldwide, respectively [1, 2]. WHO estimates that daily there are more than a million new cases of STIs acquired; and that each year, one out of twenty adolescents contract an STI [1].

STIs especially among young people, individuals from 10 to 24years, have been shown to be associated with long-term reproductive consequences such as infertility, Pelvic Inflammatory Disease (PID), and increased risk of acquiring and transmitting HIV [1]. Ectopic pregnancies, abortions, stillbirths and congenital infections are some negative consequences on pregnancy [1]. Viral STIs such as HPV have been associated with increased risk of malignancies such as cervical cancer, and HSV (mainly type 2) increase susceptibility to HIV in mature epidemics [3–5]. Studies have shown HIV accounts for higher morbidity and mortality within the younger population especially women, and about seventeen percent of adolescent deaths are attributed to HIV [6, 7].

For Higher Learning Institutions (HLIs), STIs affect students’ health and well-being, may lead to drop-outs, missing classes, emotional distress, STIs complications and even HIV/AIDS [8, 9]. Most students at HLIs have the freedom from parents/ guardians’ control, tend to form new social networks and engage in sexual relationships [9–11]. Sexual activities that have been reported among university students include first sexual encounters, premarital sex and transactional sex as well as engaging in sex under the influence of alcohol and/or other substances of abuse [9–13]. A number of studies on sexual practices among young adults in SSA show suboptimal levels of preventive behaviors against STIs [9–12]. Literature demonstrates that while majority of students believe that they are at low or no risk to STIs/HIV, they were actually found to be at high risk after assessing their sexual behavior practices [13]. Evidence also showed low proportion of HLI students use condoms or used condoms at last sex, and have concurrent multiple sexual partners [9–15]. Despite reports of a decrease in risky sexual behaviours among young people globally, no significant effect has been observed in STIs/HIV incidence [16].

In Tanzania, syndromic case management (SCM) based on diagnosis of specific symptoms and clinical signs of STIs is recommended for diagnosis and management of STIs at the first level of contact in health settings [17]. Prevention, control and proper management of STIs for a focused public health action requires accurate information on STI burden; and this is well emphasized on the WHO Global Health Sector Strategy on STIs 2016–2021 [18]. Estimation of the STI burden globally and especially in resource-poor settings is challenged by availability of diagnostics, limited studies on STI occurrence and sub-optimal STIs surveillance systems [18]. Limited numbers of studies have been conducted among university students in SSA, and of these, very few quantified prevalence of HIV/STIs [9–11, 13] while others assessed STIs only as a report of “*presence of genital discharge*” [10]. The few sero-behavioural studies done among HLIs students quantified mainly HIV and did not include other STIs. This study aimed to describe the prevalence of and risk factors associated with Herpes Simplex Virus-type 2 (HSV-2), *Chlamydia trachomatis* (CT), *Neisseria gonorrhoeae* (NG), Syphilis and HIV infection among young adults aged 18-24years attending HLIs in Mbeya-Tanzania.

## Methodology

### Study design, setting and population

We carried out a cross-sectional study among 18–24 year old students attending Higher Learning Institutions (HLIs) in Mbeya region, Tanzania. Mbeya is located within the Tanzanian Southern Highlands Zone and has two international borders with Zambia and Malawi. Southern Highlands regions are among regions with a high HIV prevalence in Tanzania; Mbeya recorded a HIV prevalence of 9.3% and ranks third [19]. The region consists of a youthful population [20] and had 6 HLIs registered by the Tanzania Commission for Universities (TCU) during the study period, namely St. Augustine University of Tanzania (SAUT), Mbeya Teofilo Kisanji University (TEKU), Mzumbe University–Mbeya University College (MUMCo), Mbeya University of Science and Technology (MUST), Tanzania Institute of Accountancy (TIA) and Open University of Tanzania (OUT).

Each HLI was invited to participate and inclusion criteria for participants included being a bona fide student at any of the 6 HLIs in any year of study or course, Tanzanian, aged 18–24 years and agreeing to provide written informed consent prior to all study-related procedures. Exclusion criteria included students attending short-term courses (< 6 months) or elective students. An invitation to participate was sent to each HLI and all institutions agreed to take part but the Open University of Tanzania had only older students (> 24 years) and was excluded.

Sample size estimation followed proportions assessment in cross-sectional studies using random sampling adjusted for non-response. We estimated a minimum sample requirement of 494 participants, based on HSV-2 prevalence of 26.7% from a study done in Kenya [21]. Considering challenges in measuring precise sexual behaviour, HSV-2 infection due to its high infectiousness, similar routes of transmission and the likelihood of being acquired before other STIs, is used here as a marker of high risk sexual behaviour to determine and map out populations at an increased risk for other STIs.

A complete list of all students aged 18–24 years registered at each HLI, as well as their contact information, was requested and obtained from the Academic Registrars’ offices. The total number of students (categorised by sex) from each HLI was determined using a probability proportional to size following the varying number of students at each HLI. Using Stata software version 14 for Windows (*Statacorp*, *4905 Lakeway Drive*, *College Station*, *Texas 77845 USA*), potential participants were selected using a computerized random number. Each selected eligible student was informed about the study through a phone call, and students that agreed to participate were requested to report to the data collection point within their respective campuses. If the phone number was not reachable or the selected participant had no mobile phone, the class representative assisted to physically find the selected participant and a face-to-face appointment was scheduled. Each participant was verified using a student identification proof before study procedures could commence.

### Data collection methods, tools and study procedures

*Data and Sample collection*, *Clinical examination and testing*: Information on socio-demographics, sexual experience, sexual behaviours, STI knowledge and presence of STI symptoms was collected using an individual self-administered questionnaire (*S1 File*). We collected information on age when participant first experienced any act of a sexual kind (such as kissing, petting or feeling one another), sexual intercourse, and history of forced sex at sexual debut or with a regular partner. Participants completed the questionnaire using a tablet or smartphone through a web-based software Open Data Kit (ODK) or hard copy, which ever method the participant preferred. The questionnaire was developed following adoption of a number of questions from various research studies and survey questionnaires to assess sexual risk behaviours (https://www.natsal.ac.uk/media/2097/final-questionnaire_technical-report-appendix-b.pdf); and modified to relate to the Tanzanian setting. The questionnaire was sent out to 50 HLI students from a nearby region (Iringa) for pre-testing and later revised accordingly before commencing the study. This questionnaire is not yet validated.

Clinical examination procedures based on established routine clinical procedures at the Mbeya Zonal Referral Hospital (MZRH) were followed, and included assessment of symptoms related to STIs or reproductive tract infections. Participants were asked if they have experienced pain, burning or stinging sensation when passing urine, passing urine more often than usual, genital wart or lump and/or genital ulcer or sore; and for females, abnormal vaginal discharge with or without itching, unpleasant odour associated with vaginal discharge, vaginal pain during sex, abnormal bleeding between menstrual periods, bleeding after sex (not during menstrual period) and/or lower abdominal or pelvic pain (not related to menstrual period) within a month before the study. Venous blood (in a 4mls EDTA tube) and urine samples were collected from all enrolled participants following their consent to provide such samples. Blood samples were collected by a specialised nurse/clinician. Participants were instructed to collect first catch urine specimen.

Samples were processed at the College of American Pathologies (CAP) accredited NIMR-MMRC laboratory in Mbeya. Samples were transported to the laboratory in a cool container maintaining 2°C to 8°C, and reached the laboratory for processing not later than an hour after collection. Diagnosis of HIV infection was done according to the Tanzania National HIV testing algorithm for adults. A blood sample was first tested using SD Bioline HIV- 1/2 3.0 (*Standard Diagnostics Inc*.) rapid test and if the first test was reactive, a second confirmatory test, Uni-Gold HIV-1/2 (*Trinity Biotech*) rapid test was done using the same sample. Diagnosis of HIV infection was established if both rapid tests results were reactive; in case of discordant results, tests were repeated using a different sample. Virotech HSV-2 (gG2) ELISA Assay (*Virotech Diagnostics GmbH*) was used for the semi-quantitative and qualitative detection of specific IgG antibodies to HSV-2 in participants’ serum samples. Results were reported in Virotech units (VE), the ratio of the absorbance units of the positive control to participants’ serum sample against a cut-off control multiplied by 10. Test results were positive if the measured VE were above 11.0, borderline if between 9.0 and 11.0, and negative if VE were below 9.0. Wampole Impact RPR (*Alere*), a non-treponemal test, was used for the rapid detection of active Syphilis infection. The test is based on the detection of reagin, present in serum from infected individuals, by using carbon-particle cardiolipin antigen. Reactive samples cause flocculation with the carbon particles and appear as black clumps against a white background; non-reactive samples appear to have a uniform light grey colour. In Standard Operating Procedure (SOP), titres of the serum samples that were reactive by RPR would be determined to an endpoint and later tested for antibodies to *Treponema pallidum* by using *Treponema pallidum* Passive Particle Agglutination Assay (*Fujirebio Diagnostics Inc*.). NG and CT were tested from fresh or stored up to 24-hours urine samples through point of care (POC) test–Xpert^®^ CT/NG assay using two modules GeneXpert® Instrument System (*Cepheid*). Urine samples were stored at 2°C to 8°C at the NIMR-MMRC laboratory before analysis.

### Data management and statistical analysis

Data quality control and assurance was ensured by smart checks for incomplete or ambiguous responses integrated in the web-based data collection tool (ODK). Questionnaires were reviewed for completeness at the end of each day by the Research Assistant. Data were imported in an Excel database, and thereafter cleaned and analysed using statistical software Stata version 14 for Windows (*Statacorp*, *4905 Lakeway Drive*, *College Station*, *Texas 77845 USA*). Data were summarised descriptively using percentages and/or proportions for categorical variables, mean and respective measure of dispersion for numerical variables. The primary outcome measure for this study was diagnosis of any of the STI tested, as a binary variable defined by a positive/reactive test result “*yes*” or negative/nonreactive test result “*no*”. Invalid or undetermined results were excluded from the analysis. STI prevalence and risk factors analysis was restricted to participants who reported to have had a sexual encounter or sex, and any participant who tested positive to any of the STIs tested even if he/she did not report to have had a sexual encounter or sex. In univariate logistic regression analysis, we explored independent risk factors to STIs. Odds ratios (ORs) with 95% confidence intervals were calculated and all statistical tests were performed using two-sided tests at 5% level of significance. All variables, whether significant in the univariate analysis or not, were included in the multivariate logistic analysis.

### Definition of variables

STI knowledge was assessed using a set of 17 questions and each correct response was given a single mark. For each participant, STI knowledge was ranked based on the score of correct responses to either Excellent (above 75%), Moderate (45%-75%) or Poor (below 45%); however, this variable was re-grouped due to low frequencies in the “Excellent” group. Non-marital, sexual relations within 6 months before the study with a partner(s) of at least 5years either older or younger than the participant were self-reported. Oral sex practices included any act of putting one’s mouth on a penis, vagina or genitals. Sexual orientation was evaluated based on participants’ response whether heterosexual (attracted to opposite sex only), homosexual (attracted to same sex only), bisexual (attracted to men and women) or preferred not to say. Residency reflected a place of permanent or long stay of the participant prior enrolment to a HLI within Mbeya.

### Ethics

Ethical approval was requested and granted by the National Health Research Ethics Committee (*Reference Number NIMR/HQ/R*.*8a/Vol*.*IX/3092*), Mbeya Medical Research and Ethics Review Committee (*Reference Number SZEC-2439/R*.*A/V*.*1/07*) and Kilimanjaro Christian Medical College Research Ethics and Review Committee (*Reference Number 2405*). A participant’s study identification number was used in the questionnaire; and only the Principal Investigator and Research Assistant had access to any information that could directly identify the participant. Participants’ right to refuse participation or withdrawal was exercised, and no further information was collected following refusal to participate or withdrawal.

Once laboratory results were available and confirmed by the study team, participants were informed through a phone call and requested to visit the data collection point to receive their results as well as post-test counseling. Results were communicated back to all participants. Treatable STIs were managed following the WHO and Tanzanian STI treatment guidelines, and all participants were counseled further on the disease risk factors. Participants diagnosed with either CT or NG were requested to inform their sexual partners to also get treatment, or if they agreed, they accompanied them to the data collection point for treatment and counseling. Participants diagnosed with HIV, received post-test counseling and were referred to a HIV Care and Treatment Centre that the participant preferred for ART initiation and further management.

## Results

### Response

Study participants were enrolled between March 2019 and January 2020. A total of 632 randomly selected students from 5 HLIs in Mbeya aged 18–24 years were contacted by the study team, and of those, 504 agreed to participate. The remaining 128 students either could not be reached or were not willing to participate in the study. No further information was collected following refusal to participate. Among the 504 enrolled participants, four female participants did not consent to an HIV test and one male participant did not consent to a NG/CT test.

### Characteristics of the participants

Socio-demographic and sexual characteristics of the 504 participants are shown in Table 1. The mean age was 21.5 years (SD 1.7), 57.3% were males; majority were single (93.9%), in their first or second year of University (78%), financially supported by a parent/guardian (86.3%) and not permanent residents within Mbeya (69.8%). Participants who reported to have had sex were 446 (88.5%), and their mean age at sexual debut was 18.4 years (SD 2.2). Bisexual and homosexual practices were reported by male (32/287) as well as female (42/217) participants.

**Table 1:** Socio-demographic and sexual characteristics of the participants (N = 504).

Variable	n (%)
Sex	
Female	217 (43.1)
Male	287 (56.9)
Age	
18–19	85 (16.9)
20–24	419 (83.1)
Mean (SD) age of participants	21.5 (1.7)
Religion^*^	
Christian	427 (84.7)
Muslim	63 (12.5)
Marital status	
Single	473 (93.9)
Married	12 (2.4)
In a relationship/cohabiting	19 (3.8)
University year	
First	223 (44.3)
Second	170 (33.7)
Third	108 (21.4)
Fourth	3 (0.6)
Employment status	
Un-employed	497 (98.6)
Employed	7 (1.4)
Financial source	
Parent/Guardian	435 (86.3)
Sponsorship/Well-wisher	37 (7.3)
Self/ Others	32 (6.4)
Permanent residency within Mbeya^*^	
No	352 (69.8)
Yes	151 (30.0)
Participants who have had sex	446 (88.5)
Mean (SD) age at sexual debut	18.4 (2.2)
Sexual orientation	
Heterosexual	244 (48.4)
Bi/Homosexual	74 (14.7)
Prefer not to say/Not applicable^ 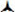 ^	186 (36.9)

^*^ Variable had missing or non-reported data

^
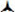
^ Not applicable reflects participants who have not had a sexual encounter

### Prevalence of laboratory confirmed STIs among participants who have had sex

CT infection was the most common STI (11%; CI 8.6–14.7), followed by HSV-2 (6.1%; CI 4.4–9.1). The prevalence of HIV and NG infection was low, 0.7% (CI 0.2–2.1) and 1.1% (CI 0.5–2.8) respectively. We collected information on whether a participant has ever been told by a doctor/healthcare professional that they had an STI (HIV was included) prior to the study; the three participants who tested positive to HIV did not know their HIV status. None of the participants were diagnosed with Syphilis. A total of 8/446 (1.8%) participants were diagnosed with two infections (four had CT and NG, three had HSV and CT, one had HIV and CT), and of these, 5 were females. Prevalence of any STI was 17%, higher among females than males (21.1% versus 14.1%). With exception of NG infection, all other STIs were higher among females than males, Table 2.

**Table 2:** Prevalence of laboratory confirmed STIs among participants who have had sex (N = 446).

STI tested	Men (N = 260)	Women (N = 186)	Total
	n (%, 95% CI)	n (%, 95% CI)	n (%, 95% CI)
HIV^Þ^			
Positive	0 (0.0)	3 (1.6, 0.5–5.0)	3 (0.7, 0.2–2.1)
Negative	260 (100.0)	179 (98.4, 95.0–99.5)	439 (99.3, 97.9–99.8)
*Herpes Simplex Virus*-Type 2^Þ^			
Positive	11 (4.5, 2.5–7.9)	16 (9.0, 5.6–14.2)	27 (6.4, 4.4–9.1)
Negative	236 (95.5, 92.1–97.5)	162 (91.0, 85.8–94.4)	398 (93.6, 90.9–95.6)
*Chlamydia trachomatis* ^Þ^			
Positive	24 (9.3, 6.3–13.5)	25 (14.4, 9.9–20.5)	49 (11.3, 8.6–14.7)
Negative	235 (90.7, 86.5–93.7)	149 (85.6, 79.5–90.1)	384 (88.7, 85.3–91.4)
*Neisseria gonorrhoeae* ^Þ^			
Positive	4 (1.5, 0.6–4.1)	1 (0.6, 0.1–4.0)	5 (1.1, 0.5–2.8)
Negative	255 (98.5, 95.9–99.4)	173 (99.4, 96.0–99.9)	428 (98.8, 97.2–99.5)
Any STI^†^			
Yes	36 (13.8, 10.1–18.6)	40 (21.5, 16.1–28.1)	76 (17.0, 13.8–20.8)
No	224 (86.2, 81.4–89.9)	146 (78.5, 71.9–83.9)	370 (83.0, 79.2–86.2)

^†^ Any STI: either HIV, HSV-2, CT, or NG

^Þ^ Some samples were either invalid, undetermined or consent was not given

### Factors associated with any STI among participants

Presence of STI symptoms one month prior to the study was reported by 123/446 (27.6%); and symptoms were reported by 16/49 (32.7%), 3/5 (60%) and 11/27 (40.7%) of participants diagnosed with CT, NG and HSV-2, respectively. Reported symptoms included pain, burning or stinging sensation when passing urine, presence of a genital ulcer or sore among participants diagnosed with HSV-2, NG and/or CT; and specifically for female participants, unpleasant odour associated with vaginal discharge, and vaginal pain during sex among participants diagnosed with HSV-2 and/or CT, abnormal vaginal discharge with or without itching, and abnormal bleeding between menstrual periods among participants diagnosed with CT only. In univariate analysis, Sex, inconsistent condom use in the past 4weeks, report of oral sex, sexual orientation (bisexual/homosexual) and having a sexual partner with an age-difference of at least 5years (either older or younger) were significantly associated with presence of an STI, Table 3. In the multivariate analysis, Sex, inconsistent condom use in the past 4weeks and sexual orientation (bisexual/homosexual) remained significant. Females had 2-times higher odds of having an STI than males (adjusted OR 2.41; 95% CI: 1.22–4.76). Likewise, participants who reported inconsistent condom use had almost 3-times higher odds of having an STI than others (adjusted OR 2.70; 95% CI: 1.08–6.75).

**Table 3:** Factors associated with presence of an STI among participants (N = 446).

Variable	N	Any STI	Univariate OR	p-value	Multivariate OR	p-value
		n (%)	(95% CI)		(95% CI)	
Sex						
Male	260	36 (13.9)	1			
Female	186	40 (21.5)	**1.70 (1.04–2.80)**	**0.035**	**2.41 (1.22–4.76)**	**0.012**
Age group						
18–19	70	13 (18.6)	1.13 (0.59–2.19)	0.711	1.28 (0.59–2.76)	0.528
20–24	376	63 (16.8)	1			
Age of sexual debut*						
≤ 15 years	44	8 (18.2)	1.09 (0.48–2.46)			
> 15 years	360	61 (16.9)	1	0.837	1.88 (0.75–4.73)	0.180
History of concurrent multiple sexual partners						
Yes	120	51 (15.6)	1.42 (0.83–2.42)	0.198	1.18 (0.60–2.35)	0.629
No	326	25 (20.8)	1			
Level of STI Knowledge						
Poor	228	36 (15.8)	1			
Moderate & Excellent	218	40 (18.4)	1.20 (0.73–1.96)	0.473	1.64 (0.88–3.06)	0.117
Report of STI symptoms in the past month						
Yes	123	25 (20.3)	1.36 (0.80–2.31)	0.256	1.16 (0.60–2.23)	0.655
No	323	51 (15.8)	1			
Condom use in the past 4weeks						
Yes, used every time	218	32 (14.7)	1		**2.70 (1.08–6.75)**	
Yes, used sometimes	44	13 (29.6)	2.44 (1.15–5.15)	0.020	**1.79 (0.94–3.38)**	**0.033**
Not used/not reported	184	31 (16.9)	1.18 (0.69–2.02)	0.551		**0.074**
Sex under influence of alcohol/marijuana/ illegal drugs^*Δ*^						
Yes	31	7 (22.6)	1.56 (0.64–3.78)	0.325	0.77 (0.25–2.41)	0.653
No	381	60 (15.8)	1			
Report of oral sex ^*ø*^						
Yes	115	28 (24.4)	**1.90 (1.12–3.21)**	**0.017**	1.04 (0.51–2.11)	0.910
No	331	48 (14.5)	1			
Sexual orientation						
Heterosexual	244	32 (13.1)	1			
Bi/Homosexual	74	21 (28.4)	**2.62 (1.40–4.92)**	**0.003**	**2.34 (1.07–5.09)**	**0.032**
Prefer not to say/not reported	128	23 (18.0)	1.45 (0.81–2.60)	0.212	1.54 (0.75–3.14)	0.239
Having a sexual partner with an age-difference of at least 5years (either older or younger)						
Yes	249	56 (22.5)	**2.57 (1.48–4.45)**	**0.001**	**1.91 (0.98–3.72)**	**0.056**
No	197	20 (10.2)	1			

^*^ variable had missing or non-reported data

^Δ^ analysis restricted to participants who only reported to have had used alcohol or marijuana or illegal drugs

^ø^ Include also those who reported having oral sex while under the influence of drugs/alcohol

## Discussion

Prevalence of HSV-2, CT, NG and HIV infection among young adults attending HLIs in Mbeya was found to be 6.1%, 11%, 1.1% and 0.7%, respectively. Syphilis was not detected in any of the samples collected. Females had the highest prevalence of any STI (either HIV, HSV-2, CT and/or NG*)*. Females, participants who used condoms inconsistently, practiced oral sex, bisexual/homosexual behaviours and had a sexual partner with an age-difference of at least 5years (either older or younger) were independently associated with presence of an STI.

HSV-2 which causes Genital herpes and Genital Ulcer Disease is a highly infectious and common STI with reported strong correlation to sexual practices, such as inconsistent condom use and oral sex, in different groups. HSV-2 infection, clinically and sub-clinically, has also been associated with increasing HIV transmission [1] among various groups and due to its high infectiousness, it is usually acquired before HIV. In this study 6% of young people had HSV-2, lower from what has been observed in other studies among adolescents/young people [21, 22]. This could be a reflection of the study participants, whereby attainment of higher level education can positively determine sexual behaviour against STIs [23]. However, many studies on HSV-2 have focused among high at-risk groups such as female sex workers, pregnant women and HIV infected individuals. HSV-2 infection has similar routes of transmission as HIV, and prevalence of HSV-2 increases with age (possibly due to duration of sexual activity span and number of sexual partners). From our HSV-2 prevalence findings, we can deduce that young adults in HLIs maybe at an increased risk for HIV as well. Given that HSV-2 is a recurrent infection and many cases may be asymptomatic, the need to educate students at HLIs on general STIs and their asymptomatic nature cannot be over emphasized. Serological etiological tests can be done periodically to help in monitoring trends of infections over time.

From our findings, the most prevalent STI was CT (11%). Chlamydia is often asymptomatic with serious consequences especially for young participants who would have never known they had this infection. Complications following untreated CT infection include chronic PID, infertility, ectopic pregnancy, prostatitis, urethral strictures, and other adverse pregnancy outcomes [1]. In Tanzania, due to inadequate laboratory infrastructure and the high costs of laboratory tests, SCM is used [17]; however, following the asymptomatic nature of CT infection, it becomes challenging for SCM to correctly identify and treat infected individuals [17, 24]. This leads to persistence of the infection which increases chances of complications as well as numbers of infected individuals who may act as “*carriers*” further transmitting STIs to their sexual partners and/or within their community because asymptomatic individuals are not likely to seek treatment. Counseling to young people diagnosed with an STI on the importance of partner notification and treatment need significant emphasis to stop transmission and re-infection. Furthermore, it is indeed about time to consider ways of introducing point-of-care screening tests for STIs such as Chlamydia, especially for younger age groups, given the asymptomatic nature of the infection.

Asymptomatic or cases with mild symptoms are often missed by syndromic management and underrate the magnitude of STIs. It is therefore important to give information and education to young people on key symptoms of STIs, its consequences and promote timely medical care seeking behavior. In this study, 27.6% of participants reported an STI symptom within a month prior to the study and only 48.9% of the participants had moderate to excellent knowledge. Awareness and knowledge about STIs is an essential component to behaviour change; misconceptions about level of perception of risk to STIs may result from ignorance as a result of lack of comprehensive sexual education from an early age (example secondary or high school). Prevention campaigns need to consider switching gears to give other STIs, which predispose young adults to HIV, the deserved attention because HIV has received a lot of attention over the years and appear to have camouflaged other STIs. It is also paramount that Information, Education and Communication (IEC) packages for health promotion campaigns emphasize the importance of prevention through multidisciplinary strategies to minimize exposure risk. In Tanzania, young people face challenges and barriers of cost, provider stigma and judgment when trying to access SRH services [25, 26]; and therefore, health facilities may not be suitable in identifying and treating young people infected with an STI. Young adults and other at-risk groups need targeted education on safer sexual practices, such as proper, consistent and negotiation of condom use as well as reduction in number of concurrent sexual partners.

In this study, the prevalence of HIV was low (0.7%) and only observed among female participants. The low HIV prevalence from our findings is similar to findings from sero-behavioural studies conducted among a number of Universities in Kenya, Uganda and Tanzania [9–11]. It appear that even though university students are sexually active and have substantial risk for STIs, little evidence was found of substantial increased HIV risk among them. In a population-based HIV impact assessment conducted in Tanzania, disparities were noted in HIV prevalence between males and females such that HIV prevalence was double or higher among young female adults aged 15–19 years, 20–24 years as compared to males in comparable age groups [27]. Similar to our findings, such differences were observed among female compared to male participants. Vulnerability to STIs increases among young women for a number of reasons, such as biological, social and/or cultural. Immune responses being not yet fully developed and the lack of enough protective antibodies as compared to older women, elevate their exposure risk to infections [28]. Socio-cultural factors may include lack of sexuality education, poor access to SRH services, stigma, sexual violence and incompetency to negotiate sexual matters [8]; the latter being of concern in cases where age difference between sexual partner is distinct.

Having sexual partners with an age difference of 5 years or more was significantly associated with occurrence of an STI in this study. Other studies have reported on university students having more than a single sexual partner or sexual relations with older partners [9, 11, 12, 29], which successively increase the chance of acquiring and further transmitting an STI or HIV. Female students tend to have older partners compared to male students who have partners who are usually younger [12]. The likelihood of unprotected sexual acts is higher among young adults with multiple sexual partners [29] and in sexual relations with a partner of pronounced age-difference (cross-generational relationships). The latter is another significant fuel of the STI/HIV epidemic, because older sexual partners are probably HIV infected already [30]. Multiple sexual relations and/or cross-generational sex among students in resource limited SSA may be driven by socio-economic factors, such that young adults from low socio-economic backgrounds may be tempted to practice risky sexual behaviours so as to match up to their financial needs while at campus or act on “peer-pressure”. The younger partner in a cross-generational relationship has limited ability to negotiate sexual matters as compared to the older partner that often bears the financial and other social benefits. This increases the STI/HIV risk as most times older male partners prefer younger women believing they are "clean" of STIs and hence would not suggest protected sex [31]. Studies have shown highly educated people have more sexual partners than those with a lower education level [29]; and strict monogamous type of relationships in HLIs are said to be uncommon [32]. IEC initiatives among young educated adults should consider and influence the ability to discuss and agree on safe sex and sexual matters, avoid coercion and peer-pressure as well as realising one’s worth.

The HLI community is a mobile population, such that staff and students migrate from their primary locations to either work, teach or study to a particular region where the university is located temporarily or permanently. Staff and students interact with existing surrounding communities at the particular location/region in many ways. Sexual relationships, commercial or otherwise, have been reported and a number of students opt to have sexual partners outside the universities [10]. An assessment of universities in Tanzania noted reasons for this being easy access, cheap and the relationships were not as stressful as those with fellow students [10]. Acknowledging the high HIV prevalence within Mbeya region [19] and the prevalence of STIs noted in our findings as well as the risk factors associated with presence of an STI, it is important to consider the HLI community as an at-risk population as well as a potential driver of the infection to the community at large. It is also important country wide to maintain surveillance on prevalence of STIs periodically especially among young adults, because understanding the epidemiology of STIs among at-risk groups may spearhead evidence-based policies and guidelines to control the STI problem. HLIs on the other hand need to work with district and Community Based Organisations (CBOs) working in the field of STIs/ HIV and consider periodic screening campaigns within campus facilities.

### Strengths and limitations

Findings from this study will add on body of knowledge and literature of the existing limited numbers of studies conducted among university students which quantify prevalence of HIV/STIs. The study measured precise sexual behaviour by combining self-reports with biological markers used as proxy for risky sexual behaviour so as to identify those at a higher risk for STIs. This controlled for social-desirability response bias. Participants may tend to provide socially desirable responses and not the true practice of their sexual behaviours so as to portray good low risk behaviour.

Measuring precise sexual behaviour is challenging as such behaviours occur in private and respondents may feel uncomfortable opening up openly about high-risk sexual behaviours. The downfall of using biological markers includes scenarios where those practicing risky behaviours and/or their partners remain uninfected and may not be included as having practiced such. Further, biological markers provide information on just the presence of risky sexual practices but not on the magnitude or intensity of it. The study findings may have been affected by the probability of STI transmission which depend on number of those infected within the given population, the number of their sexual partners and contacts per partner, as well as the likelihood of meeting a partner who has an STI [33]. The test used for diagnosis of HSV-2 infection detected presence of specific IgG antibodies to HSV-2 from serum samples, and therefore, presence of acute infections may have been missed.

In Tanzania, quite a significant number of children and adolescents do not attain post-primary/secondary education and the number is even lower to HLIs; therefore our study findings may not be generalizable to many young people who have not reached higher education level. This “*elite*” group of people may differ from young adults who are out of universities/colleges.

Information on circumcision, PrEP and current contraception use was not collected and this is acknowledged as a potential study limitation.

## Conclusion

Presence of data on STI/HIV burden among at-risk population is crucial to providing a platform which can guide prevention campaigns and surveillance strategies for a streamlined public health initiative. Even though the prevalence of HIV among young adults in HLIs is found to be low, other STIs such as Chlamydia and HSV-2 which are commonly asymptomatic are of concern. National periodic STI prevalence monitoring is important to curb the STI problem, since control through SCM faces a number of challenges in correctly identifying and treating infected individuals. Young adults attending HLIs could be an important group that needs attention and recognition that is pivotal in transmission of STIs considering their risk, mobile nature of the HLI community and the noted interaction with their existing surrounding communities/general population.

### Recommendations

IEC campaigns targeted to young adults, especially those at HLIs, should emphasize on the importance of STI prevention, primary and secondary, through multidisciplinary strategies. Health education packages need to focus on exposure-risk minimization. Targeted IEC packages should focus on advocating safer sexual practices such as proper, consistent and negotiation of condom use as well as reduction in number of concurrent sexual partners and cross-generational sexual practices; education on the asymptomatic nature of many of the STIs and possible symptoms which may occur, consequences of untimely treatment of STIs, partner notification and treatment and promote timely health care seeking behavior. IEC among young educated adults need to focus on influencing their ability to negotiate safe sex, avoiding coercion and peer-pressure as well as realising their worth.

Relevant stakeholders need to look into barriers such as the level of knowledge to STIs and understanding the burden that could challenge young adults’ access to SRH services so as to quickly identify and treat those who may be infected with an STI. Funding institutions that have invested heavily on HIV prevention campaigns should consider giving similar recognition to other STIs for a streamlined outcome.

HLIs need to acknowledge the presence and risk of STIs within their community and work jointly with other stakeholders for periodic screening campaigns, condom use promotion and SCM within campus facilities.

It is also crucial nationally to invest in accumulating knowledge on the epidemiology of STIs, maintaining surveillance over time especially among at-risk groups and lead evidence-based policies and guidelines to regulate the STI problem.

## Supporting information

S1 File Self administered questionnaire. (PDF)
